# Immunoregulatory mechanism studies of ginseng leaves on lung cancer based on network pharmacology and molecular docking

**DOI:** 10.1038/s41598-021-97115-8

**Published:** 2021-09-14

**Authors:** Zao-Hui Li, Dan Yu, Nan-Nan Huang, Jun-Kai Wu, Xiao-Wei Du, Xi-Jun Wang

**Affiliations:** 1 Pharmacy College, Heilongjiang University of Chinese Medicine, 24 Heping Road, Harbin, 150040 China; 2 Jilin Agricultural Science and Technology University, 77 Hanlin Road, Jilin, 132101 China

**Keywords:** Drug discovery, Diseases

## Abstract

*Panax ginseng* is one of the oldest and most generally prescribed herbs in Eastern traditional medicine to treat diseases. Several studies had documented that ginseng leaves have anti-oxidative, anti-inflammatory, and anticancer properties similar to those of ginseng root. The aim of this research was to forecast of the molecular mechanism of ginseng leaves on lung cancer by molecular docking and network pharmacology so as to decipher ginseng leaves' entire mechanism. The compounds associated with ginseng leaves were searched by TCMSP. TCMSP and Swiss Target Prediction databases were used to sort out the potential targets of the main chemical components. Targets were collected from OMIM, PharmGKB, TTD, DrugBank and GeneCards which related to immunity and lung cancer. Ginseng leaves exert its lung cancer suppressive function by regulating the several signaling proteins, such as JUN, STAT3, AKT1, TNF, MAPK1, TP53. GO and KEGG analyses indicated that the immunoreaction against lung cancer by ginseng leaves might be related to response to lipopolysaccharide, response to oxidative stress, PI3K-Akt, MAPK and TNF pathway. Molecular docking analysis demonstrated that hydrogen bonding was interaction's core forms. The results of CCK8 test and qRT-PCR showed that ginseng leaves inhibit cell proliferation and regulates AKT1 and P53 expression in A549. The present study clarifies the mechanism of Ginseng leaves against lung cancer and provides evidence to support its clinical use.

## Introduction

According to the IARC, lung cancer was the malignant tumor with the highest mortality rate due to the poor prognosis^1^. Surgery is the first choice for lung cancer treatment, but most patients have already reached the middle and late-stage and have lost the best surgery chance when diagnosed. Therefore, radiotherapy and chemotherapy became the main clinical methods for treating lung cancers. However, conventional therapies have large toxic side effects, which interrupts most patients in completing an effective radiotherapy and chemotherapy cycle. Scholars have turned their attention to the development and utilization of traditional Chinese medicine, striving to develop safe and effective lung cancer adjuvant drugs.

Ginseng, dried roots of *Panax ginseng *C. A. Mey., is a precious Chinese herb with a long history of medicinal use. It was first published in "Shen Nong's Materia Medica" and listed as the top grade. Modern chemical and pharmacological studies have found that the extracts of ginseng leaves contained more ginsenosides than the roots and showed potent anticancer activity^2–4^. However, most of the researches are the anti-cancer effects of individual components of ginseng leaves. The polysaccharides in ginseng leaves inhibit tumor metastasis through activating macrophages and NK cells. Ginsenosides enhance the immune response of innate immune cells such as dendritic cells, phagocytes and NK cells by affecting the immune system and kill virus infects cells and tumor cells, regulate cytokines, complement and other immune molecules to produce immunoregulatory effects^5–7^. There is no report on the overall mechanism of multi-component and multi-target of ginseng leaves in the immunomodulation treatment of lung cancer. Traditional Chinese medicine has the characteristics of multiple components and multiple targets, with definite curative effect, relatively few adverse reactions, and low price^8^. It has attracted more and more attention from pharmaceutical workers. However, the features of traditional Chinese medicine (TCM) have caused bottlenecks in the study of its mechanism of action, affecting its worldwide application and promotion.

In 2007, Professor Hopkins, a British scholar, put forward the concept of "network pharmacology" based on network biology. Its core idea is to reveal the mechanism of drug action and guide drug molecular designed by multi-dimensional, multi-pathway, and multi-target points of the interaction network of genes, proteins, and metabolites^9^. At present, a large number of reports have proved that the computer simulation such as molecular docking and network pharmacology can successfully predict the active ingredients, targets and mechanisms of TCM, save the cost of drug development, and provide new strategies for the research on the material basis and mechanism of TCM^10–12^.

**Figure 1:**
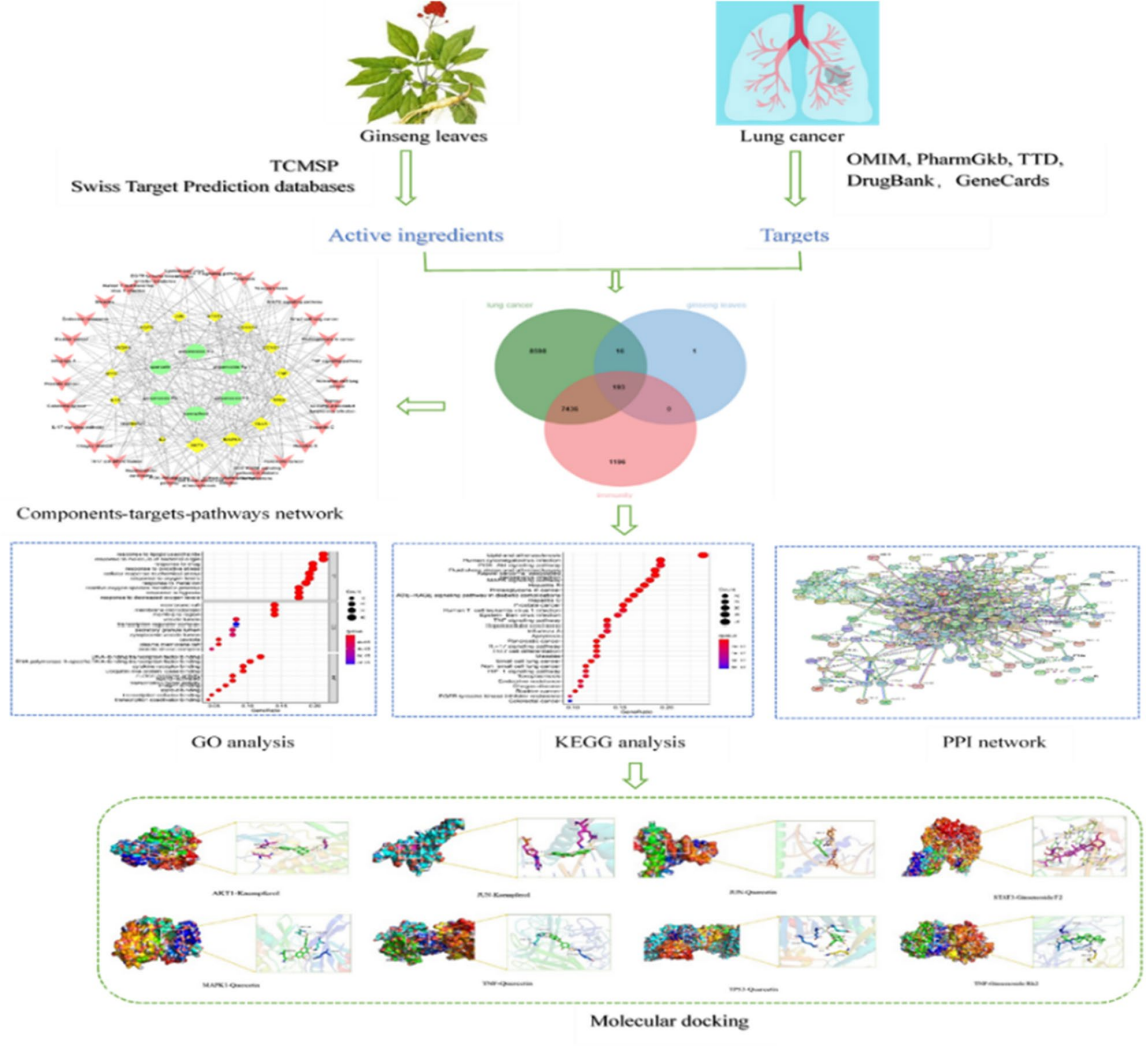
Workflow diagram of the network pharmacology-based analysis of ginseng leaves in the treatment lung cancer.

Therefore, the aim of this article was to clarify the overall mechanism of ginseng leaves in lung cancer therapy by adopting network pharmacological and molecular docking, providing new insights into the effects and mechanisms of ginseng leaves and a scientific basis for the clinical application. The work flow chart is shown in Fig. 1.

## Result

### Active ingredients and related targets of ginseng leaves

From TCMSP, one hundred and twenty-nine ingredients in ginseng leaves were acquired. Since OB and DL represent the most important pharmacokinetic properties, OB ≥ 30% and DL ≥ 0.18 were set as the limitation for the active ingredients ^13,14^. There were nine compounds meeting the parameter. As shown in the Table 1, eight compounds were finally identified as the active chemical ingredients of ginseng leaves (Table 1). According to the TCMSP database and the Swiss Target Prediction database, two hundred and ten targets that were associated with the above active chemical compounds were discovered.

**Table 1:** Core components of panax ginseng leaves.

MOL ID	Molecule name	OB	DL
MOL000359	Sitosterol	36.91	0.75
MOL000422	Kaempferol	41.88	0.24
MOL005344	Ginsenoside rh2	36.32	0.56
MOL000098	Quercetin	46.43	0.28
MOL003975	Icosa-11,14,17-trienoic acid methyl ester	44.81	0.23
MOL006733	Ginsenoside-F2	37.03	0.25
MOL005338	Ginsenoside Re	4.27	0.12
MOL011401	Ginsenoside Rg1	9.03	0.28

### Screening mutual targets of drugs and diseases

**Figure 2:**
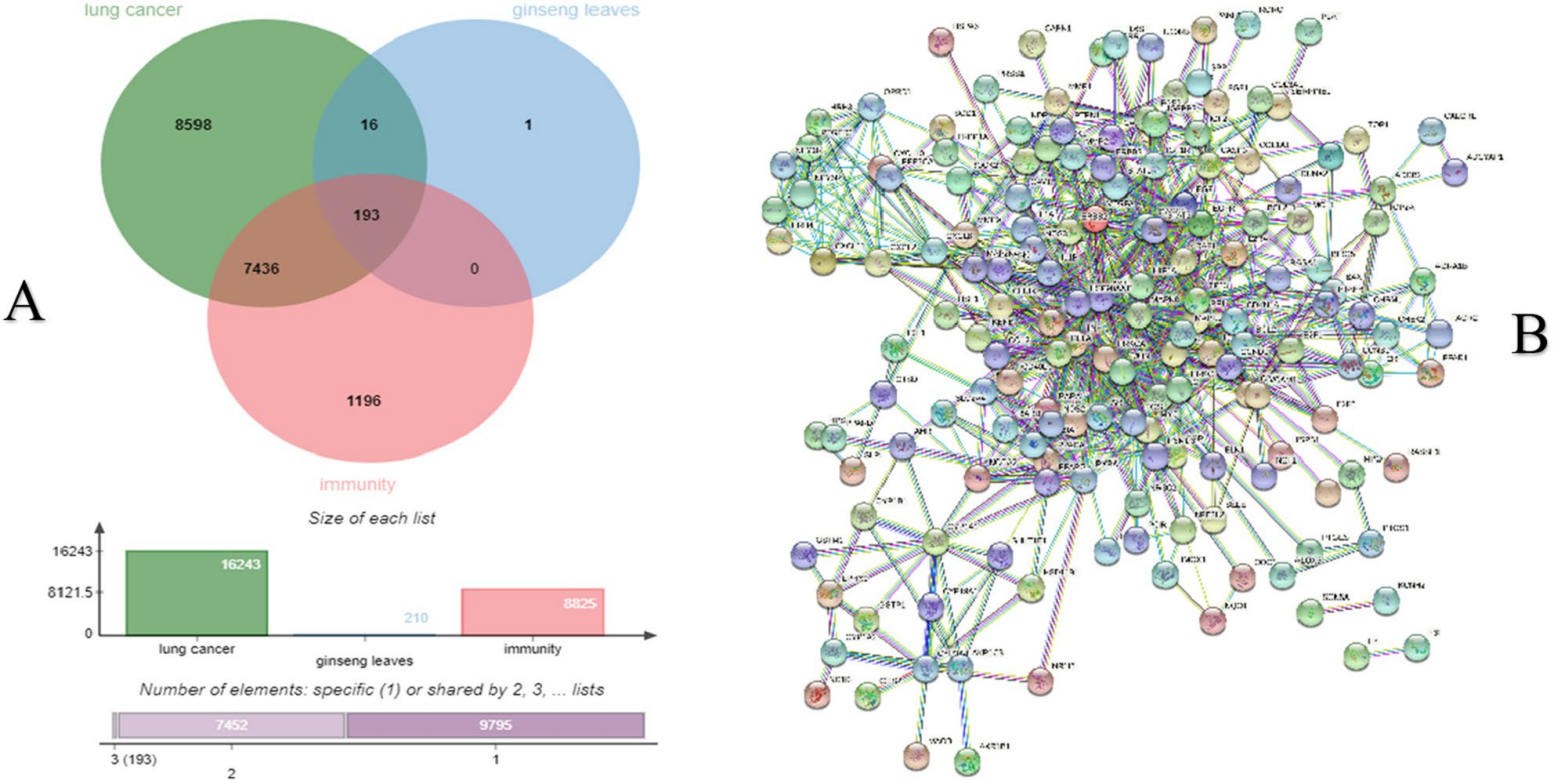
An association network of ginseng leaves targeted proteins associated with lung cancer and immunity. **(A)** Venn diagram of ginseng leaves, lung cancer and immunity targeted genes. **(B)** The network for 193 co-targeted genes/proteins had been selected as input for PPI analysis in STRING.

The core active component targets of ginseng leaves were matched with the targets of lung cancer and immunity, resulting in the selection of 193 composite targets of ginseng leaves, lung cancer and immunity (Fig. 2A) (Supplementary Table S1).

### Screening and topological analysis of core targets

**Figure 3:**
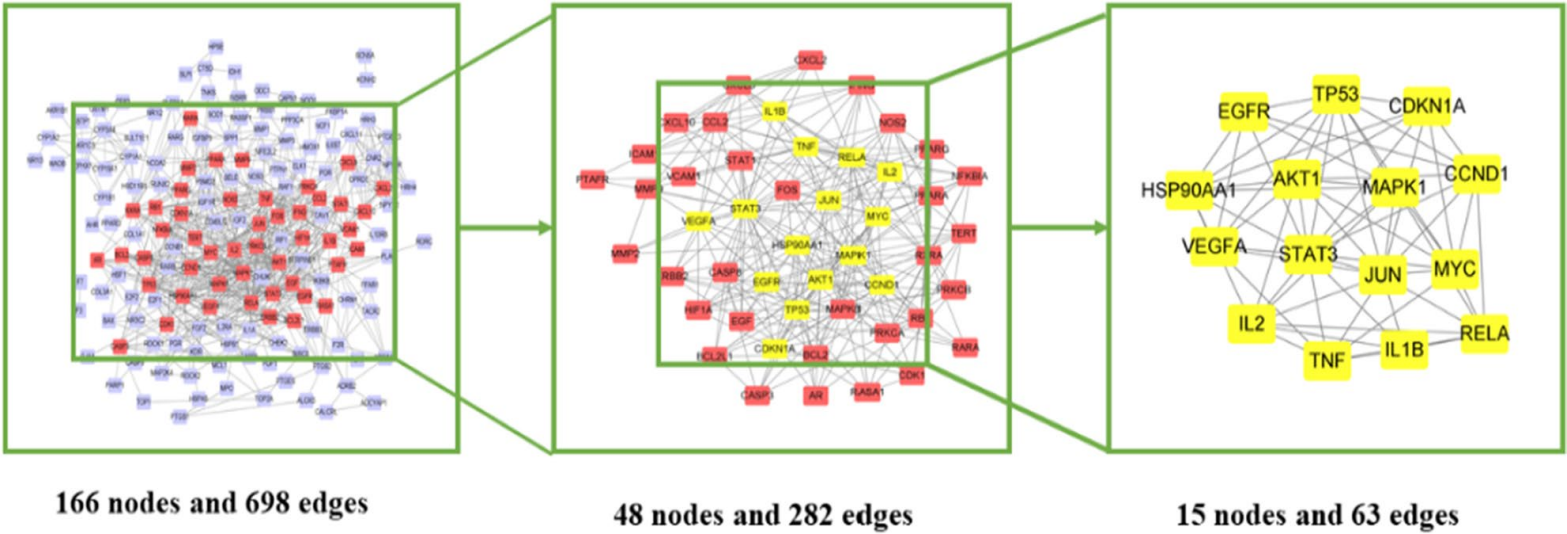
The protein–protein interaction (PPI) network. **(A)** The PPI network containing 166 nodes and 698 edges. The areas highlighted in red are represented the candidate targets ranked in the median values for the DC, BC, CC, EC, LAC, NC. **(B)** The PPI network for the candidate targets ranked in the median values for the DC, BC, CC, EC, LAC, NC. The PPI network containing 48 nodes and 282 edges. The areas highlighted in yellow are represented the potential targets with the median values for the DC, BC, CC, EC, LAC, NC. **(C)** The PPI network for the 15 potential targets with the median values for the DC, BC, CC, EC, LAC, NC.

The PPI of the above 193 intersection targets was obtained on the STRING platform (Fig. 2B). The PPI map was taken when the data was import into Cytoscape 3.8.0. The PPI network was built with 166 nodes and 698 edges. The interaction is represented by each edge between proteins. Each Node represents the target protein molecules^15^. Then, according to the network topology's features, the median values for the DC, BC, CC, EC, LAC, NC were used to analyse potential drug targets^16^. Fifteen highly connected nodes (degree > 10, BC > 16.3058, CC > 0.5402, LAC > 5.4444, NC > 6.3355) were confirmed as significantly related targets with Network Analyzer in Table 2 and Fig. 3. The PPI network shows the targets involved in lung cancer and immunity were HSP90AA1, JUN, STAT3 EGFR, MYC, VEGFA, CCND1, TNF, MAPK1, AKT1, RELA, CDKN1A, TP53, IL2 and IL1B which were the major targets in lung cancer.

**Table 2:** Information on 15 core targets.

Name	BC	CC	DC	EC	LAC	NC
CDKN1A	18.28429903	0.573170732	12	0.152390316	6.5	7.867424242
JUN	98.74824512	0.661971831	23	0.264033586	8.869565217	15.5701324
HSP90AA1	106.6241734	0.626666667	19	0.192736804	6.315789474	10.97384282
EGFR	38.14004426	0.5875	14	0.150455698	6	8.272352647
TNF	145.8416338	0.652777778	22	0.212447509	7.090909091	14.26343186
RELA	106.8717418	0.618421053	20	0.199681893	6.5	11.37552225
TP53	110.8916672	0.661971831	23	0.246520162	8.608695652	17.18136716
STAT3	433.9244392	0.746031746	31	0.294780642	8.322580645	24.45735784
MYC	30.3784685	0.602564103	16	0.201331109	7.75	10.39949495
VEGFA	43.6583161	0.5875	14	0.156834617	6	8.683477633
IL1B	24.65080698	0.552941176	12	0.12125846	5.833333333	7.707575758
CCND1	23.87157903	0.573170732	12	0.152351752	5.833333333	6.818181818
IL2	26.99749461	0.580246914	13	0.164281353	6	7.232323232
MAPK1	154.2495504	0.68115942	25	0.264529794	8.24	17.45260829
AKT1	100.2562622	0.643835616	21	0.227695495	7.428571429	13.18314741

### Enrichment analysis of GO and KEGG pathway

**Figure 4:**
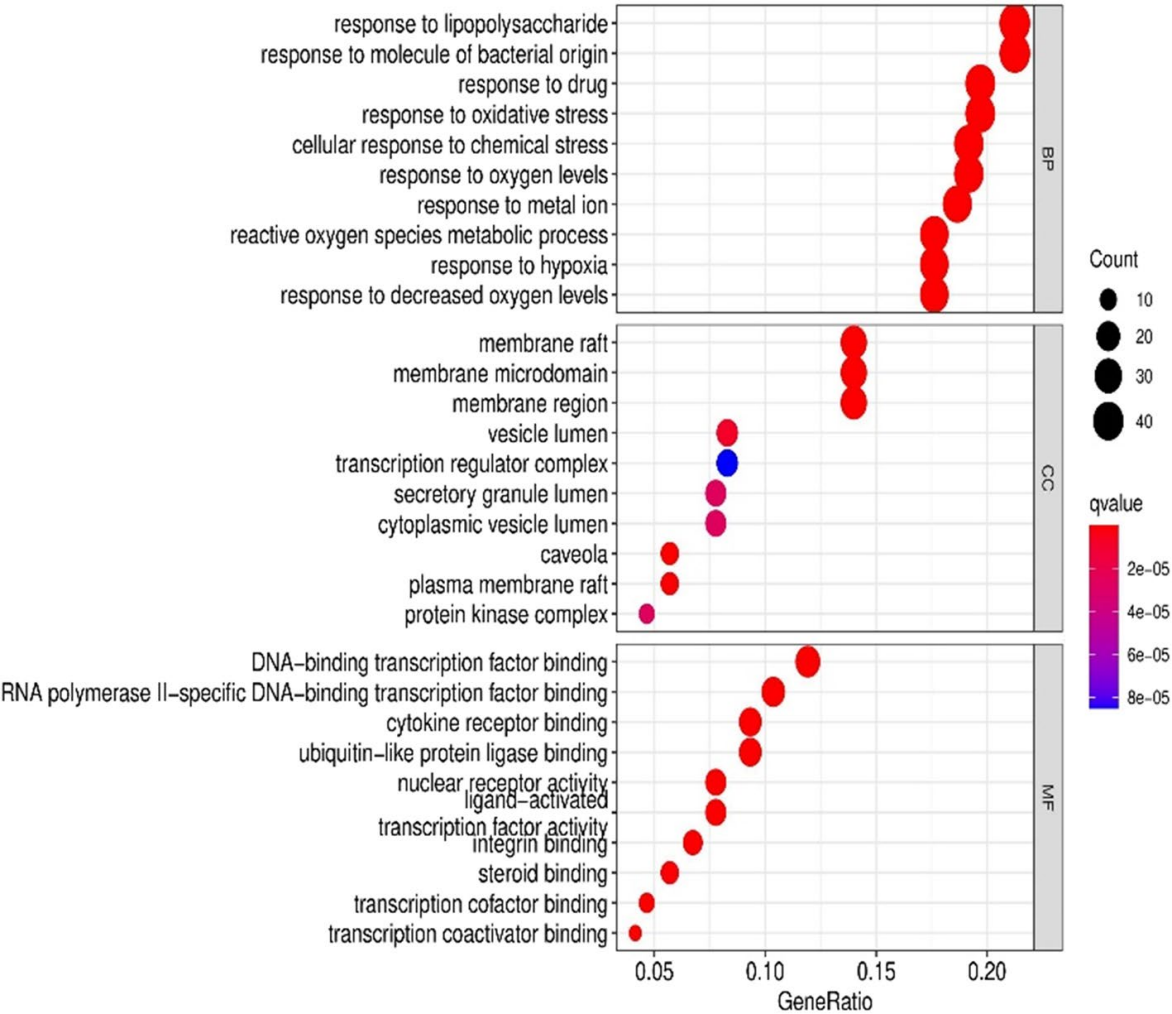
The GO enrichment analysis of core nodes. Including cellular components, molecular functions, biological processes.

GO functional enrichment analysis of key targets was performed relying on Bioconductor package in R software. The GO terms were screened according to the P (P < 0.05, Q < 0.05) value. There were 2363, 45, and 181 GO terms related to biological processes, cell components, and molecular functions, respectively (Fig. 4). As showed in Fig. 4, the top 10 biological processes were related to lipopolysaccharide, nutrient levels, oxygen levels, etc. For cellular components, the targets were enriched in membrane raft, membrane microdomain, transcription regulator complex, etc. Molecular Functions analysis revealed including DNA-binding transcription factor, ubiquitin-like protein ligase, cytokine receptor, oxidoreductase activity, etc.

**Figure 5:**
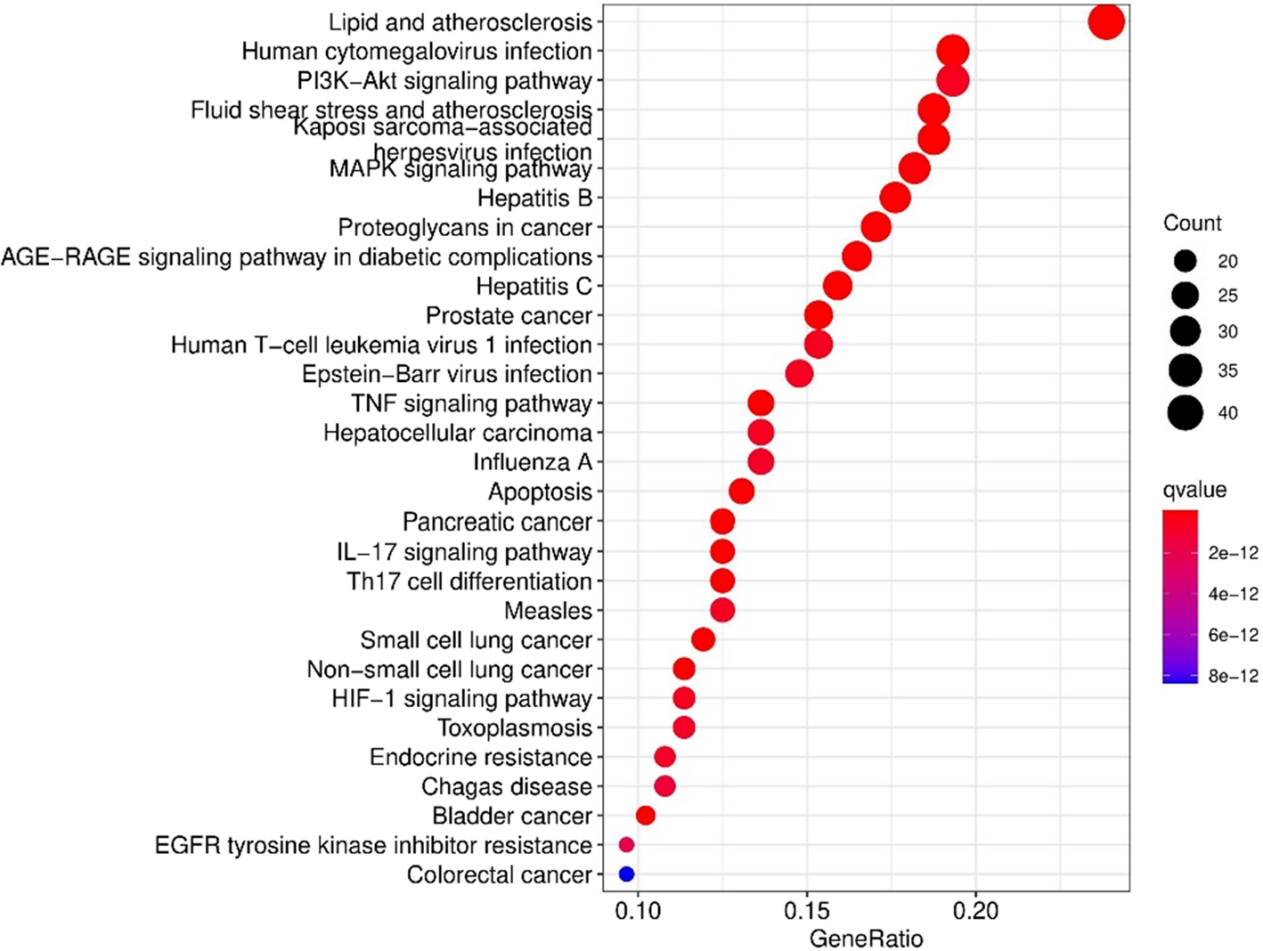
The top 30 pathways for KEGG enrichment analysis of the targets of ginseng leaves.

The KEGG signal pathway enrichment analysis contained 164 pathways. It screened the first 30 pathways related to Human cytomegalovirus infection, PI3K–Akt pathway, MAPK signaling pathway, Small cell lung cancer, Non-small cell lung cancer (NSCLC), Proteoglycans in cancer, Prostate cancer, TNF signaling pathway, Hepatocellular carcinoma, IL-17 signaling pathway, Apoptosis, etc. (Fig. 5) ^17–19^. Comparing with the gene ontology and KEGG analysis outcomes, genes encoding proteins targeted by ginseng leaves should play a role in PI3K–Akt pathway, MAPK signaling pathway, TNF signaling pathway, Small cell lung cancer, Non-small cell lung cancer, apoptosis. These pathways involved cytokine receptor.

### Active ingredients-shared key targets-signal pathway network diagram construction

**Figure 6:**
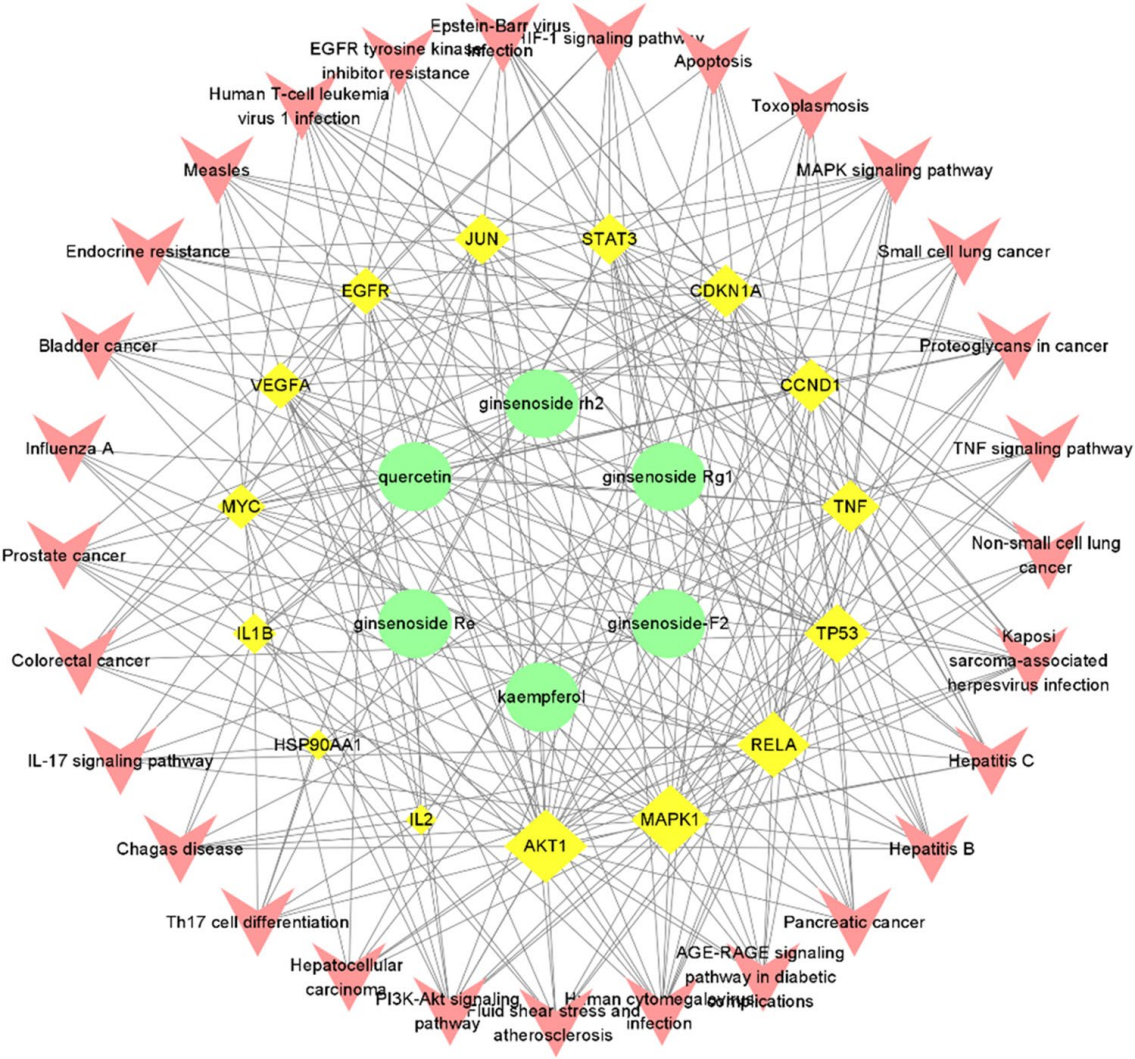
The “active components-target genes-pathways” network.

The component-target-channel network diagram was formed to holistically make clear ginseng leaves' mechanism in Lung cancer immunity through Cytoscape 3.7.2. (Fig. 6). In the figure, the green Ellipse represented the ingredients in the drug, the yellow Diamond represented the targets of the ingredients, and the red V shape represented the pathways enriched in the targets. It demonstrated that each active compound could act upon multiple targets, each target can also respond to multiple active ingredients, and each target can have an effect on multiple pathways.

### Key active ingredients and core target molecular docking results

**Figure 7:**
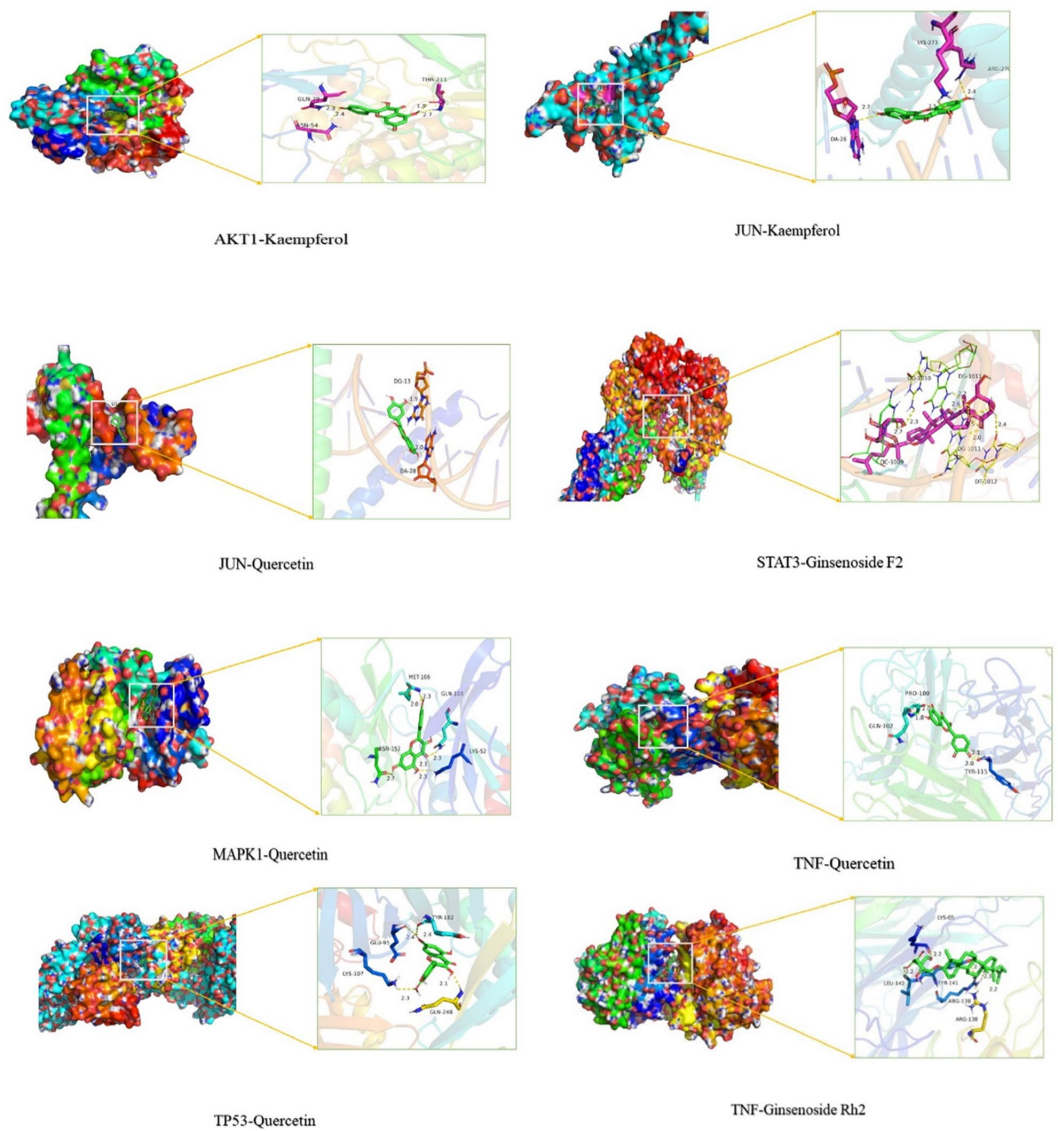
Detailed target-compound interactions.

The top six-core targets of degree in the PPI network were molecularly docked with their corresponding active ingredients. The docking protein data were downloaded from the PDB database, which was STAT3 (PDB ID 6qHD), MAPK1 (PDB ID 2OJI), TP53 (PDB ID 7BWN), JUN (PDB ID 5T01), TNF (PDB ID 2TUN) and AKT1 (PDB ID 4ejn). The 3D structure was imported into AutoDock software and docked with the active compound. The top six core targets in the PPI network and their corresponding small molecule drug ligands were docked using AutoDock Vina and R 4.0.2 software (Table 3). The smaller the binding energy is, the better the ligand can bind to the protein. Mode 1 with the lowest binding energy was visualized using Pymol software (Fig. 7). The primary forms of interaction were hydrogen bonding and π–π stacking. This result indicated that their combination might play an important role in the treatment of lung cancer with ginseng leaves.

**Table 3:** Docking scores of the active ingredients of ginseng leaves with their potential targets.

Targets	Compounds	Affinity (Kcal/mol)
STAT3	Ginsenoside-F2	−10.5
AKT1	Kaempferol	−9.5
TNF	Quercetin	−9.4
TP53	Quercetin	−8.9
JUN	Kaempferol	−8.9
JUN	Quercetin	−8.6
TNF	Ginsenoside rh2	−8.4
MAPK1	Quercetin	−8.2

### Cytotoxicity on human lung adenocarcinoma cells line A549

**Figure 8:**
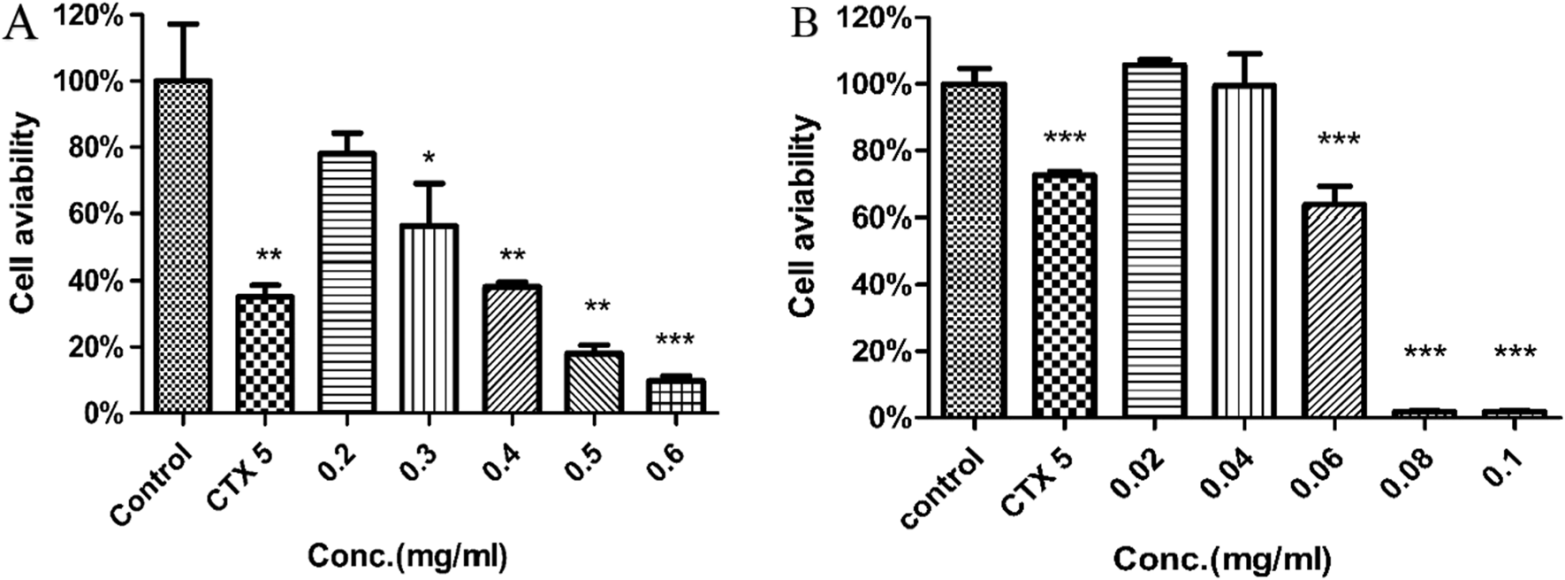
Ginseng leaves extract and F2 significantly decreases the viability of human lung adenocarcinoma cells. **(A)** Effects of the ginseng leaves extract on the viability of A549 cells. **(B)** Effects of the F2 on the viability of A549 cells.

A549 was significantly decreased after 24 h exposure to ginseng leaves extract and F2 in a concentration-dependent manner(Fig. 8A,B). Cytotoxic effect of ginseng leaves extract and F2 were confirmed against human Lung Adenocarcinoma Cells line A549.

### Effect of ginseng leaves extract on the gene expression of AKT, STAT3 and P53 in A549

**Figure 9:**
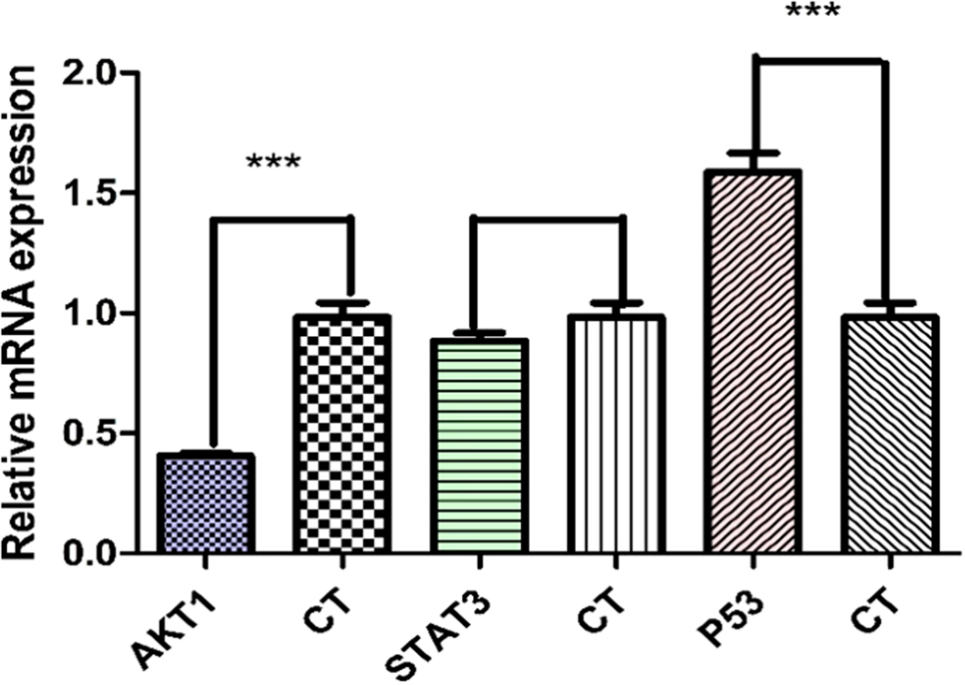
The mRNA expression levels of AKT1, STAT3, P53. Compared with the control group, the expression levels of AKT1 and P53 mRNA have significant difference in the treatment group (***p < 0.0001).

The mRNA expression levels of AKT1 and P53 were significantly different between the blank control group and ginseng leaves extract group (p < 0.05).Meanwhile, P53 was up-regulated expression, while AKT1 was down-regulated expression. Furthermore, there was no significant difference between groups in STAT3 mRNA expressions (Fig. 9).In conclusion, the results suggested that the above core targets could play important roles in ginseng leaves extract treatment of lung adenocarcinoma.

## Discussion

Lung cancer is one of the most ordinary malignancies on earth, and its high morbidity and mortality seriously threaten the health of people all over the world^20,21^. TCM exerts its anticancer effects through apoptosis induction, proliferation inhibition, metastasis suppression, multidrug resistance reversal and immune function regulation. Moreover, TCM can ameliorate patients' quality of life^22,23^. A number of data show that ginseng leaves can inhibit tumor migration and invasion by regulating the crosstalk between tumor-related macrophages and non-small cell lung cancer, and improve the body’s immune function^24–27^. Therefore, this paper researched the action mechanism from the point of immunomodulatory on lung cancer by the integrity of ginseng leaves.

In this paper, the core active components in ginseng leaves were collected through data mining. The mechanism of inhibiting lung cancer through immune regulation was explored microscopically by network pharmacology. In the Venn diagram where the active ingredients of ginseng leaves intersect with diseases, there were 209 intersecting targets between drugs and lung cancer, and 193 intersecting targets between drugs and immunity. One hundred and ninety-three composite targets of ginseng leaves, lung cancer and immunity are the same as the immune intersection targets. The results showed that 92% of the treatment of ginseng leaves for lung cancer could be achieved by immune regulation.

BP analysis results showed that core active components were involved in reactive oxygen species metabolic process, response to lipopolysaccharide, response to oxidative stress, etc. Oxidative stress is characterized by the imbalance between reactive oxygen species (ROS) production and the capacity of the antioxidant system. Oxidative stress injury results in lipid peroxidation. The final product of lipid peroxidation can cause subsequent pathological consequences through the rearrangement of peroxyl radical, especially in the lungs^28^. Oxidative stress had been well documented to play a major role in cancer. Oxidative damage to DNA caused by ROS is thought to be one of the causes of cancer. Lipopolysaccharide (LPS) is a crucial component of the Gram-negative bacteria cell wall^29^. A change in the state or activity of the body caused by lipopolysaccharide stimulation. In vivo, endothelial cells, epithelial cells, monocytes and macrophages can be activated through the cell signal transduction system. Synthesizing and releasing various cell-stimulating factors, stimulating the body's active defense, cause a series of inflammatory reactions, and exert the early immune response effect. Studies have confirmed that LPS can enhance the invasion and metastasis abilities of lung cancer and other malignant tumor cells^30,31^.

In the KEGG enrichment, Pathways associated with lung cancer and immunity and more enriched in 14 potential targets were PI3K–Akt pathway, MAPK signaling pathway, Non-small cell lung cancer.HSP90AA1, EGFR, MYC, VEGFA, CCND1, MAPK1, AKT1, RELA, CDKN1A, IL2 and TP53 targets are enriched in the PI3K–Akt pathway. This pathway is activated by various types of cellular stimuli or toxic insults to regulates fundamental cellular functions such as transcription, translation, proliferation, growth, and survival. PI3K/AKT pathway's deregulation is involved in lung tumorigenesis and it has been connected with high-grade tumors and advanced disease^32^. Deregulation of this pathway proceeds through a variety of mechanisms including upstream activation by tyrosine kinase receptors of PI3K, PIK3CA amplification as well as mutations in KRAS, PI3K or AKT^33^. The MAPK signaling pathway regulates the cell cycle, and the increase in its activity is positively correlated with the proliferative capacity of tumor cells, disease development and poor prognosis^34^. JUN, EGFR, MYC, VEGFA, TNF, MAPK1, AKT1, RELA, IL1B and TP53 are enriched in the MAPK signaling pathway. Studies have found that this effect may be related to the induction of Th1 cells to produce IFN-γ and promote cell proliferation^35,36^.

In the network topology analysis, the active ingredients of ginseng leaves have a greater possibility of acting on the immune regulation of lung cancer through HSP90AA1, JUN, STAT3 EGFR, MYC, VEGFA, CCND1, TNF, MAPK1, AKT1, RELA, CDKN1A, IL1B, IL2 and TP53. TP53 and JUN are important tumor suppressor genes and are new directions for gene targeted therapy of cancer^37,38^. TP53 strictly regulates the start of the cell cycle and can repair damaged DNA in time. When genes are mutated and signal pathways are abnormal, the cell cycle is out of control and certain genetic damages cannot be repaired and mutated, accelerating the progression of the tumor^39^. Studies have verified that the up-regulation of the HSP90AA1 gene reduced the body’s immune surveillance and resistance to foreign substances, and involving DNA self-repair capabilities^40^. STAT3 is a transcription factor activated by various external stimuli and can induce cell growth, differentiation and survival^41^. It is mentioned that STAT3 activity is enhanced in up to 50% of all human tumors^42^. It is known that the activation of STAT3 in many malignant tumors is related to tumor proliferation, invasion, metastasis, and resistance to chemotherapy and radiotherapy^43,44^. In NSCLC, STAT3 is continuously activated in 22–65% of cases, and STAT3 activation is linked to poor prognosis, proliferation, and chemotherapy resistance^45–48^. EGFR as a transmembrane glycoprotein, which is one of the four members of the tyrosine kinase receptor ErbB family^49^ is considered to be the driving gene of the tumor in the development of NSCLC^50,51^. VEGFA is a considerable target for anti-tumor therapy, and its expression is negatively associated with the differentiation and prognosis of lung cancer^52^. CCND1, the most important member of the cyclin family, is currently considered an oncogene and is overexpressed in human esophageal cancer, head and neck cancer, and breast cancer. The overexpression of CCND1 is considered an early incident in diverse carcinomas^53^.

Tumor necrosis factor TNF is called death ligand and can bind to extracellular death receptor to initiate the apoptosis program^54^. TNF-α interacts with TNFR1 and TNFR2 receptors and activates downstream intracellular signaling pathways. TNF-α induces both apoptosis and necrosis of cells through the TNFR1^55^. MAPK1 is chiefly involved in the regulation of cell proliferation, differentiation and apoptosis^56^. RelA transcription factors is a member of the NF-κB family of transcription factors and control gene expression in response to stimuli such as inflammation^57^. The abnormal expression of Myc is also associated with lung cancer, which was significantly overexpressed in more than 70% of NSCLC^58^. Early Myc activity is causally related to cancer mainly through its ability to drive tumor cell proliferation to participate in biosynthesis, cell metabolism; angiogenesis, invasion and metastasis^59^. CDKN1A plays a key role in the progression of the cell cycle and regulates both G1/S and G2/M checkpoints. CDKN1A, as an oncogene, promotes cancer cell proliferation by inhibiting apoptosis^60,61^. Interleukin-*1β* (*IL*-*1β*) is an inflammatory cytokine that belongs to the *IL*-1 family^62^. IL-1β is an effective driver of tumor progression. It is highly expressed in metastatic non-small cell lung cancer and drives tumor growth, invasion and metastasis^63^. Interleukin-2 (IL-2) is a cytokine signaling molecule necessary for the differentiation, growth and proliferation of T lymphocytes. It has been shown to improve the survival rate of patients with non-small cell lung cancer^64^. IL2 stimulates the proliferation and activation of immune cells with anti-tumor activity^65^. In summary, multiple targets and pathways are closely related to the occurrence, development, and prognosis of lung cancer, confirming the characteristics of multi-component, multi-target and multi-channel treatment of ginseng leaves.

In the component-target-pathway network, four core active components and 15 key potential targets are involved, all of which play an important part in anti-lung cancer through immune regulation. Molecular docking results indicate that the components had effective free energy against key targets. These findings verify the reliability of the active ingredients screened by network pharmacology and their interaction with lung cancer and immunity targets. Ginsenoside F2 and STAT3 have the best affinity. It has been noted that F2 induces apoptosis by causing an accumulation of ROS and activating the ASK-1/JNK signaling pathway^66^. F2 induces apoptosis of breast cancer by activating the intrinsic apoptotic pathway and mitochondrial dysfunction. However, the potential anti-cancer effect of ginsenoside F2 in lung cancer cells has not been appraised^67^. In this study, F2 inhibits the proliferation of A549 by the CCK8 experiment, but its mechanism of action is not yet understood. Further study will be made later on the basis of this finding.

Based on the research strategy of network pharmacology, this article integrates various database information and uses molecular docking for preliminary verification, and clarifies that the core components of ginseng leaves have multi-component and multi-target mechanism characteristics in inhibiting lung cancer through immunomodulation. However, Chinese medicine is not a single set of chemical components. Data mining and network pharmacology are based on the existing big data to make reasonable predictions about the internal mechanism of ginseng leaf inhibiting lung cancer through immune function. In the later stage, the above mentioned potential medicinal ingredients, target points, and mechanism of action need to be experimentally verified to provide a more scientific basis for the development and utilization of ginseng leaves and clinical research.

## Methods

### Screening of core active ingredients and targets

All the chemical constituents of Ginseng Folium were retrieved based on the Traditional Chinese Medicine Systems Pharmacology Database and Analysis Platform (TCMSP)^68^. According to the pharmacokinetics (ADME) parameters in the TCMSP database, oral availability (OB) ≥ 30% and drug-like activity (DL) ≥ 0.18 were set to screen the core active ingredients. Search for the active ingredients of ginseng leaves in the "Chinese Pharmacopoeia (2020)", and add the important active ingredients initially excluded by TCMSP as candidate active ingredients. TCMSP database and Swiss Target Prediction databases^69^ were used to retrieve the gene targets for active ingredients. Obtained targets were then mapped to the UniProt database for normalization.

### Disease-associated gene mining

Targets related to lung cancer and immunization were collected from the OMIM database, PharmGkb database, TTD database^70^, DrugBank database and GeneCards database^71^. The gene name's standardization and definition of the species as "human" were executed using the UniProt database. The Venn diagram of the active components, lung cancer targets and immune targets was drawn by using the bioinformatics platform Jvenn^72^.

### Protein–protein interaction network construction and analysis

The potential targets were formed by STRING database to acquire the targets-PPI network. Selection parameters were set to “*Homo sapiens*” for species and the confidence level was set at 0.900 for the minimum required interaction score. The PPI network was then visualised by the Cytoscape software. The network analyser plugin in Cytoscape was used to calculate topological parameters in the network. Finally, the key immune regulatory target of ginseng leaves suppressing lung cancer was obtained.

### Gene ontology (GO) and the Kyoto encyclopedia of genes and genomes (KEGG) enrichment

Enrichment analysis of Potential Target Gene for the core drug group Ontology (GO) Enrichment and KEGG pathway enrichment were performed using Bioconductor cluster profiler^73^, org.Hs.eg.db and DOSE of R 4.0.2. Pathways were ranked according to the number of molecules in pathways with a cut-off value (p < 0.05).

### Network construction

The compound-target-pathway network was constructed using Cytoscape 3.7.2. The topological properties were analysed using the Network Analyzer plug-in for Cytoscape to confirm the key components and targets^74^.

### Molecular docking makes sure the interaction between targets and key compounds

The target protein structure was obtained from the RCSB PDB database. The 2D structure of ginseng leaves' active substance was sought by the PubChem Database ^75^. ChemBio Draw 3D and Autodock Tool were applied to optimize the structure of key compounds and targets, including 3-dimensional chemical structures creation, energy minimization, and format transformation^76^. PyMol was used to process the protein, including getting rid of the ligands, rectifying protein structure, and doing away with water ^74^. Docking was completed by R 4.0.2 software and Autodock Vina, and the molecules with the lowest binding energy in the docking conformation were selected to observe the binding effect by matching with the original ligands and intermolecular interactions.

### Cell culture

A549 cells were obtained from the American Type Culture Collection and were plated in RPMI 1640 medium with 10% fetal calf serum and penicillin with streptomycin. Cells were all kept at 37 °C, with 5% CO_2_.

### Cytotoxicity

Cytotoxicity was assayed using CCK8, taking ginseng leaves extract, ginsenoside F2 and cyclophosphamide as the different experimental groups and A549 cells as the blank control group. A549 cells were plated in 96-well plates in 100 μL of medium containing 10% heat-inactivated FBS. After 24 h, the cells were treated with cyclophosphamide, different concentrations of ginseng leaves extract, F2 and incubated for an additional 24 h. At the indicated time points,10 μL CCK8 reagent was added to the culture medium 1 h before analysis. Optical density (OD)450 values were measured using a microplate reader.

### Gene expression using quantitative real-time PCR

In this trial, ginseng leaves extract was used as the different experimental groups, and A549 cells as the blank control group. After being placed into a six-well plate, the A549 cells were add to culture medium containing best concentrations of ginseng leaves extract and then cultured for 48 h. Next, Total RNA, was isolated utilizing Trizol reagent (Invitrogen), whose concentration and purity were detected using SPECTROstar Nano Microplate Reader (BMG Labtech). According to the manufacturer’s protocol, the RNA was reverse-transcribed into cDNA using the extracted RNA sample as a template. The primer sequences were designed in the laboratory by Sangon Biotech (Sangon, China) based on the mRNA sequences obtained from the NCBI database and were purchased from Beijing Huada Gene and Biological Company, China (Table 4). Real-time PCR was then performed using an Applied Biosystems QuantStudio 5 Real-Time PCR system. Data reported as fold mRNA expression (2^−ΔΔCt^) and was normalized to β-actin.

**Table 4:** The mRNA sequences of the targets obtained from the NCBI database.

Gene name	Forward primer (5′–3′)	Reverse primer (5′–3′)
AKT	TGACCATGAACGAGTTTGAGTA	GAGGATCTTCATGGCGTAGTAG
STAT3	TCGGCTAGAAAACTGGATAACG	TGCAACTCCTCCAGTTTCTTAA
P53	TTCCTGAAAACAACGTTCTGTC	AACCATTGTTCAATATCGTCCG

### Statistics

The statistical analysis was performed by the Student’s t test using GraphPad Prism 5 software. P ≤ 0.05 value between groups was considered to be statistically significant.

## Supplementary Information

Supplementary Table S1.
