# Supplementary material for: Immunoregulatory mechanism studies of ginseng leaves on lung cancer based on network pharmacology and molecular docking
Source: Sci Rep. 2021 Sep 14;11:18201. doi: 10.1038/s41598-021-97115-8 (PMC8440634; doi:10.1038/s41598-021-97115-8)
Supplement: Supplementary file 1 — Supplementary Table S1. [file 41598_2021_97115_MOESM1_ESM.docx]

| CYP3A4 | CAV1 | BCL2 | KDR | PGR | CYP19A1 | VCAM1 | HSD11B2 | CHRM1 | CAPN1 |
| --- | --- | --- | --- | --- | --- | --- | --- | --- | --- |
| NR1I2 | MMP1 | HSPB1 | BIRC5 | MCL1 | CXCL10 | PTPN1 | ROCK2 | PPP3CA | PTAFR |
| TP53 | STAT1 | MMP9 | RASSF1 | HSP90AA1 | NR3C2 | SELE | DUOX2 | TNKS | PSMG1 |
| EGFR | RAF1 | MMP2 | BCL2L1 | PRKCA | MMP3 | PLAT | NCF1 | TACR2 | HRH4 |
| CHEK2 | NFE2L2 | CYP1B1 | CASP9 | COL1A1 | RUNX2 | F2R | SULT1E1 | PTGES | NPEPPS |
| ERBB2 | RARB | MAPK1 | NQO1 | CCNB1 | FGF1 | CLDN4 | ELK1 | FKBP1A | CNR2 |
| RB1 | PLAU | AR | MAPK8 | SERPINE1 | PRKCB | CXCL2 | PTGER3 | COLQ | HRH3 |
| TNF | PPARG | CASP3 | IL2 | SOD1 | CTSD | HPSE | GRIN2B | EIF6 | LIPE |
| AKT1 | SPP1 | ICAM1 | IL2RA | IKBKB | F3 | SCN5A | ACP1 | PSMD3 | OPRD1 |
| TERT | ERBB3 | PTGS2 | ALOX5 | ODC1 | SLPI | PPARA | ACACA | CALCRL | NR1I3 |
| MYC | RELA | HMOX1 | E2F1 | E2F2 | PON1 | F7 | ADCYAP1 | IL10RB | FFAR1 |
| CCND1 | CCL2 | AHR | TOP1 | CHUK | LGALS3 | CYP1A2 | LGALS4 | ADRA1B | NPY1R |
| VEGFA | CXCL8 | GSTM1 | GJA1 | IL1A | THBD | AKR1C3 | AKR1B1 | MAOB | NPY5R |
| STAT3 | TOP2A | HIF1A | PARP1 | MAP2K4 | CASP1 | KCNH2 | HK2 | RORC |  |
| IL1B | IGF2 | IDH1 | ABCG2 | PRSS1 | PTGS1 | ACP3 | SLC2A4 | RARG |  |
| CDKN1A | NOS2 | JUN | IGFBP3 | ROCK1 | RASA1 | DPP4 | CXCL11 | ATP1A1 |  |
| CASP8 | IRF1 | CYP1A1 | ADRB2 | FOS | HSPA5 | NCOA2 | INSRR | LGALS8 |  |
| EGF | CD40LG | GSTP1 | CDK1 | EPHX1 | FANCF | IL6ST | SCD | POR |  |
| IFNG | NFKBIA | IGF1R | RARA | COL3A1 | HSF1 | SLC6A2 | PPARD | GCK |  |
| BAX | MPO | FGF2 | NOS3 | RXRA | PSEN2 | HSD11B1 | RUNX1T1 | CES2 |  |

Supplementary Table S1 193 composite targets of ginseng leaves, lung cancer and immunity
